# *Curvularia coatesiae* XK8, a Potential Bioadsorbent Material for Adsorbing Cd(II) and Sb(III) Compound Pollution: Characteristics and Effects

**DOI:** 10.3389/fmicb.2021.816312

**Published:** 2022-01-27

**Authors:** Zhao Di, Li Chaoyang, Zheng Mengxi, Zhao Yunlin, Xu Zhenggang, Yang Guiyan

**Affiliations:** ^1^Hunan Research Center of Engineering Technology for Utilization of Environmental and Resources Plant, Central South University of Forestry and Technology, Changsha, China; ^2^Central South Inventory and Planning Institute of National Forestry and Grassland Administration, Changsha, China; ^3^Key Laboratory of National Forestry and Grassland Administration on Management of Western Forest Bio-Disaster, College of Forestry, Northwest A&F University, Xianyang, China

**Keywords:** fungi, heavy metal pollution, biosorption, microbial remediation, response surface experiment

## Abstract

Soil heavy metal pollution is a common problem in mining areas. The soil of the Xikuangshan located in Lengshuijiang, Hunan Province, China contains various excessive heavy metals, especially antimony and cadmium. Previous studies have shown that heavy metal-tolerant microorganisms screened from mining areas have the potential to adsorb heavy metals. In this study, we screened out a cadmium and antimony tolerant fungus named XK8 from the slags collected from the Xikuangshan. Then, we explored the single and binary biosorption characteristics of Cd(II) and Sb(III) on it. In our results, the fungus XK8 was identified as *Curvularia coatesiae* XK8 by ITS sequencing analysis. Under the optimal conditions, in binary biosorption of the XK8, the main effect of the initial cadmium concentration on the cadmium removal rate of XK8 is negative, while the main effect of the initial antimony concentration, biosorption time, and initial pH on the cadmium removal rate of XK8 is positive. The initial pH has the greatest impact on the biosorption of cadmium on XK8, followed by the biosorption time; moreover, the effects of both are stronger than the coexisting ions. SAS analysis shows that under the optimal conditions, the theoretical maximum cadmium removal rate of XK8 is 100%, and the actual removal rate is 67.57%. Compared to the single biosorption with binary biosorption, the maximum biosorption capacity of XK8 for cadmium in the composite biosorption system increased to 23.6 mg g^–1^. It shows that under the background of high antimony, Sb(III) has a promoting effect on the biosorption of Cd(II) on XK8. In summary, a cadmium and antimony tolerant fungus with strong cadmium biosorption ability under the background of high antimony was screened out. It provides a potential microbial material for the bioremediation of heavy metal pollution.

## Introduction

The excessive use and irregular discharge of heavy metals have caused serious pollution in some areas and aroused widespread public concern (Wang et al., 2018). Heavy metal pollution cannot only affect the quality, structure, and fertility of the soil, but also affect the growth and development of soil microorganisms and plants, and even endanger human health through the food chain (Bhuiyan et al., 2010). In recent years, heavy metal pollution has become one of the important reasons for the decline of soil quality and human health. Therefore, it has aroused wide attention. Co-contamination of heavy metals (e.g., Cd, Pb, and Sb) is common in mining areas and sediments (Yang et al., 2018). For example, combined antimony and cadmium pollution has been reported in soils of the antimony mine area in Hunan, Guangxi, Guizhou, Yunnan, and other regions of China (Jie et al., 2020) and also the farming soils of Suszec commune in Poland (Yang et al., 2018). Excessive intake of antimony and cadmium is harmful to humans. Antimony has similar physical and chemical properties and toxicity to arsenic. Excessive antimony is carcinogenic to the human and has a longer incubation period (Shuangshuang et al., 2009; Bin, 2014). Sb(V) and Sb(III) are the main forms of antimony pollutants in the environment, and Sb(III) is about 10 times more toxic than Sb(V). Cadmium is a heavy metal element with strong biological toxicity and mobility (Shuhua et al., 2006). For people, the accumulation of cadmium affects the absorption of calcium and phosphorus and induces osteoporosis and “pain disease.” Therefore, it is necessary to remove the antimony and cadmium from the contaminated soil.

Compared with traditional heavy metal remediation technology, microbial biosorption technology has the advantages of strong biosorption ability and no secondary pollution. Normally, heavy metal-tolerant microorganisms can be screened in areas contaminated by heavy metals (Kumar and Dwivedi, 2019). These tolerant microorganisms are potential biosorbents for heavy metals. Fungus is an ideal biosorbent. It has the advantages of strong mycelium, large contact area, strong biosorption capacity, fast growth, low environmental requirements, and strong stress resistance (Kumar and Dwivedi, 2019). In the field of fungal bioremediation, filamentous and yeasts are research hotspots. The screened biosorbents are mainly concentrated in *Penicillium sp.*, *Aspergillus sp.*, *Mucor sp.*, *Rhizopus sp.*, and *Saccharomyces sp.* (Wang and Chen, 2009; Jacob et al., 2018). A large number of them have strong biosorption capacity for a specific metal, such as *P. chrysogenum* (Xu et al., 2015), *A. niger* (Tian et al., 2019), *M. roux?* (Yan and Viraraghavan, 2008), and so on. So far, in order to accelerate the promotion and application of fungal biosorbents, the biosorption of a single heavy metal ion has been transformed into the biosorption of multiple complex heavy metal ions. Xu et al. (2017) showed that when *P. chrysogenum* simultaneously adsorbed Cu^2+^ and Cr^6+^, its biosorption capacity for Cr was stronger than that of Cu; when *Cunninghamella bertholletiae* adsorbed both Cu^2+^ and Cd^2+^, its removal rate of Cd^2+^ dropped rapidly. Noormohamadi et al. (2019) showed that when *Phanerochaete chrysosporium* simultaneously adsorbs Cd^2+^ and Ni^2+^, its biosorption capacity for Cd is stronger than that for Ni. However, the study on the biosorption of complex heavy metal ions by fungi mainly focuses on cadmium, copper, lead, and chromium, and rarely involves antimony. At present, the research on the composite biosorption characteristics of antimony and cadmium is mainly concentrated in the field of non-biological materials, such as Fe–Mn binary oxide (Liu et al., 2015) and ferrihydrite (Yang et al., 2018). For ferrihydrite, it can adsorb antimony and cadmium in the form of surface ternary complexes in the binary system. Therefore, in order to screen out microbial materials suitable for soil remediation contaminated by antimony and cadmium, it is necessary to study the biosorption characteristics of fungi under the antimony and cadmium compound conditions.

In this study, we screened out a cadmium and antimony tolerant fungus under Sb and Cd contaminated conditions. Then, we explored the single and binary biosorption characteristics of Cd(II) and Sb(III) on it. The purpose is to explore the effects of different environmental factors on fungal biosorption of Cd(II) and Sb(III), and the interaction of Cd(II) and Sb(III) on the surface of fungi. It provides microbial materials for the remediation of antimony and cadmium contaminated soils.

## Materials and Methods

### Soil Samples

The area with the most serious antimony pollution in China is the Xikuangshan, Lengshuijiang, Hunan Province (111°18′57″–111°36′40″ E, 27°30′49″–27°50′38″ N), known as the “Antimony Capital of the World” (He, 2007). The antimony mineral resources in the Xikuangshan area reach 300,000 tons, accounting for about 30% of the world’s proven reserves. The annual production of antimony accounts for about 50% of China (He et al., 2012). The content of multiple heavy metals in its soil exceeds the soil pollution risk control standards of China. Among them, antimony pollution is the most serious, followed by cadmium pollution, accompanied by moderate to mild arsenic, copper, and lead pollution (Guo et al., 2014). The soil characteristics of the soil samples for Xikuangshan are shown in Supplementary Table 1.

We collected the soil samples of 0–10 cm slags from Xikuangshan. Then, the samples were passed through a 100-mesh sieve after mixing evenly, loaded in a 50 ml centrifugal tube, and stored at 4°C.

### Isolation and Identification of Cadmium and Antimony Tolerant Fungi

The isolation of fungi from the soil samples was performed by Martin’s agar medium (Xia et al., 2015; Wang et al., 2017; Huang et al., 2018). In brief, 10 g of the soil samples was suspended with 90 ml sterile water containing glass beads and shaken on a rotatory shaker at 120 rpm for 30 min. The supernatant suspension was diluted 10 times to obtain the soil suspension with dilution of 10^–2^ g ml^–1^; 100 μl of the diluted solution was spread on Martin’s agar medium with 0.5 mM Cd(II) (CdCl_2_ 2H_2_O) and incubated at 30°C for 5–7 days to obtain single colonies (Xu et al., 2017). Each colony was purified repeatedly to obtain purified fungi. To obtain high-cadmium and antimony-tolerant fungi, the purified fungi were plated on Martin’s agar medium with 5 mM Cd(II) and 7 mM Sb(III) (C_4_H_4_KO_2_Sb 1/2H_2_O), respectively (Chien et al., 2013; Mohite et al., 2018). They were also plated on pure Martin’s agar medium as the control treatment. After incubating at 30°C for 7 days, the better-growing fungus was used as cadmium and antimony tolerant fungi.

The genomic DNA extraction and molecular identification of tolerant fungi were completed by Shanghai Meiji Biomedical Technology Co., Ltd^1^. In brief, after extracting genomic DNA, the ITS region of the resistant strain was amplified by PCR using the forward primer ITS1 (5′-TCCGTAGGTGAACCTGCGG-3′) and the reverse primer ITS4 (5′-TCCTCCGTTATTGATATGC-3′) (Xu et al., 2012). The gene sequence obtained was subjected to BLAST analysis in the National Center for Biotechnology Information (NCBI). The phylogenetic tree was constructed by the neighbor joining (NJ) method in Mega 7.0 (Xia et al., 2015).

### Preparation of the Biosorbent

The cadmium- and antimony-tolerant fungi were inoculated into the center of Potato Dextrose Agar (PDA) medium (Xu et al., 2012; Xia et al., 2015), and cultured at 7 days for 28°C to form sporulation with 80% germination rate. Under sterile conditions, the colonies were cut into small pieces and transferred to 100 ml of sterile 0.85% normal saline. To eliminate mycelial fragments and agar, we shook the solution vigorously and filtered them through a three-layer sterile cheesecloth. The concentration of spore suspension was measured with hemocytometer and diluted to 10^6^–10^7^ cfu ml^–1^ (Tian et al., 2019).

In order to improve the removal capacity of heavy metals, the biosorbent selected fungi in the logarithmic growth phase, where the fungus activity was the strongest (Deng et al., 2012); 1 ml of spore suspension was added to 49 ml PDB medium in the Erlenmeyer flask (pH = 6) and incubated at 28°C and 120 rpm for 0–7 days, respectively (Xu et al., 2012; Xia et al., 2015). The fungal biomass was measured every 24 h by dry weight method and the measurement was stopped when its biomass no longer increased. We determined the logarithmic growth period according to the growth curve, and used the liquid at this time as the biosorbent (Supplementary Figure 1).

### Growth Characteristics of Fungi in the Single and Binary System of Cd(II) and Sb(III)

The growth characteristics of the tolerant fungi were evaluated in the liquid environment; 50 ml biosorbent was added to 50 ml of PDB medium containing Cd(II), PDB medium containing Cd(II) and Sb(III), and pure PDB medium, respectively. The concentration of heavy metals in the biosorption system is shown in Supplementary Table 2.

All of the treatments were incubated at 28°C and 120 rpm. After 4 days, the culture was harvested by filtration using Whatman No. 11 filter paper, and the dry weight was gathered. All the experiments were performed with triplicates.

### Biosorption Experiment in the Single and Binary System of Cd(II) and Sb(III)

The experiment used a design with two levels of initial Cd(II) (4, 6 mg L^–1^) crossed with four levels of initial Sb(III) (0, 50, 100, and 200 mg L^–1^); 50 ml biosorbent was added to 50 ml PDB medium with different heavy metal solutions, and incubated at 28°C and 120 rpm for 4 days. At the end of the experiment, the culture was harvested by filtration using Whatman No. 11 filter paper, and the dry weight was gathered. Two new fractions were obtained: the liquid media (Fraction A) and the dried mycelium (Fraction B). The pH and metal concentration of Fraction A was measured. Biomass production (dried mass) was measured of Fraction B. The biosorption capacity (q_e_) of heavy metals by the tested fungi was calculated according to the following equations (Xia et al., 2015):


(1) $$q_{e}=\frac{\left(C_{0}-C_{e}\right)V}{M}$$

(2) $$Q=\frac{C_{0}-C_{e}}{C_{0}}\times 100\%$$

where *q*_e_ is the metal biosorption in mg metal ions g^–1^ biomass (mg g^–1^), *C*_0_ and *C*_e_ are the initial and equilibrium concentration of metal ions in the solution (mg L^–1^), V is the volume of the metal ions solution (ml), M is the amount of added dry biosorbent (g), and Q is the removal rate of heavy metals (%).

### Response Surface Experiment in the Binary System of Cd(II) and Sb(III)

According to the experimental results, we select the appropriate initial cadmium concentration and initial antimony concentration for subsequent experiments to keep the pH and biomass of the solution stable after adsorption.

The response surface experiment was used to study the adsorption characteristics of fungi to antimony and cadmium ions under compound conditions. The Cd(II) and Sb(III) removal ratio was optimized with response surface methodology by using Box-Behnken design (Su et al., 2019). The Box-Behnken design was applied with 4 factors (A: initial Cd(II) concentration, B: initial Sb(III) concentration, C: biosorption time, and D: initial pH of heavy metal solution), and four response values [Y_1_: Biomass, Y_2_: pH, Y_3_: Cd(II) removal rate; and Y_4_: Sb(III) removal rate], with the following values (Choinska-Pulit et al., 2018): A: 4, 5, 6 mg L^–1^; B: 20, 60, 100 mg L^–1^; C: 1, 4, 7 d; D: 2, 4, 6. Design-except V.8.0.6.1 (Stat-Ease Inc., Minneapolis, MN, United States) was used for data analysis. All the tests were performed in triplicate.

### FTIR Analysis

The main chemical functional groups of the strain can be identified by FTIR. In addition, the samples prepared and referenced by Li et al. (2019). All the tests were performed in triplicate.

### Data Analysis

All experiments were performed with *n* equal to 3. The experimental data used SPSS 23.0 (SPSS, Chicago, IL, United States) for statistics and analysis of variance. In all analyses, the standard deviation (S.D.) represented sample variability, and the significant difference was set to *p* < 0.05.

## Results

### Identification of Cadmium and Antimony Tolerant Fungi

**FIGURE 1:**
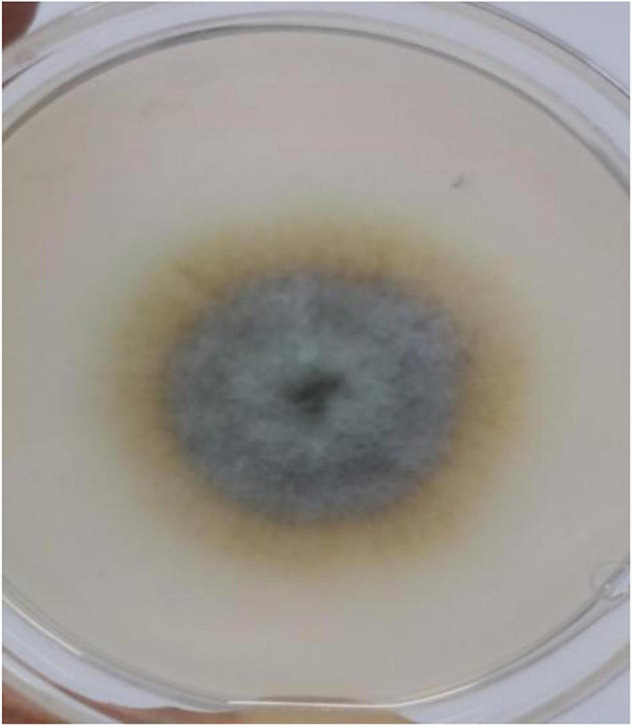
Morphological characteristics of cadmium and antimony tolerant fungi.

A cadmium and antimony tolerant strain was screened out, named XK8. The surface of XK8 was non-uniform and median rise. Its colony was large and dense, dark brown diverging outward. The edge of the colony was different from the center, and the edge was light yellow (Figure 1).

**FIGURE 2:**
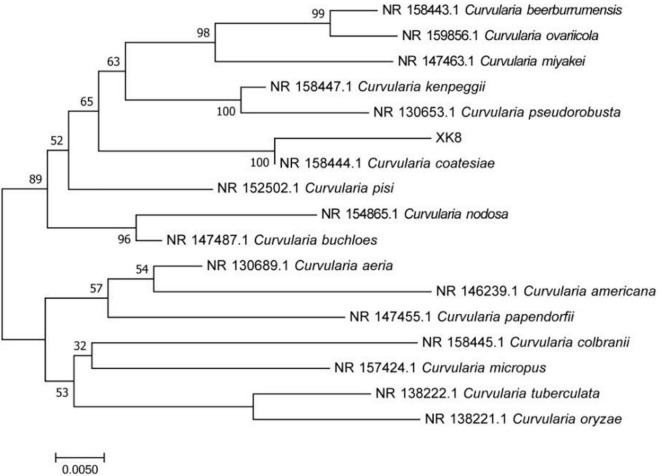
Phylogenetic tree based on 16S rRNA gene sequence. The bar represents evolutionary distance of 0.005.

The ITS rRNA sequence of XK8 was 566 bp in length. As was shown in the BLAST alignment and the phylogenetic tree, the strain was most closely related to *Curvularia coatesiae*, with a support rate of 100%. Therefore, XK8 was identified as *C. coatesiae* XK8 (Figure 2).

### Concentration of Cd(II) and Sb(III) Affecting the Growth of *Curvularia coatesiae* XK8

**FIGURE 3:**
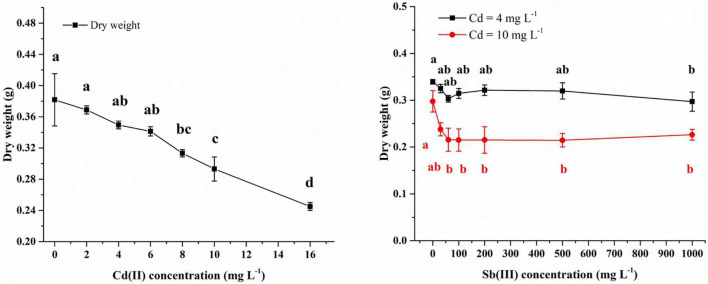
Concentration of Cd(II) and Sb(III) affecting the growth of XK8. Letters indicate results of one-way ANOVA. If the letters are completely different, the difference is significant (*p* < 0.05), otherwise the difference is not significant (*p* > 0.05).

In the Cd(II) solution, after 4 days of growth at 0–6 mg/L Cd(II), the biomass of XK8 stabilized at 0.34–0.38g. However, continued increase in the Cd(II) concentration inhibited the growth of XK8. At 16 mg/l Cd(II), the biomass of XK8 was only 0.25 g, a decrease of about 0.1 g (Figure 3).

After adding Sb(III) to 4 mg/L Cd(II) solution, XK8 grew normally when the Sb(III) concentration was 0–500 mg/L (*p* > 0.05). However, when the concentration increased to 1000 mg/L, the biomass of XK8 dropped from 0.3395 g to 0.2971 g (*p* < 0.05) (Figure 3).

After adding Sb(III) to 10 mg/L Cd(II) solution, the biomass of XK8 dropped from 0.2977 g to 0.2156 g [Sb(III) = 60 mg/L]. However, when the Sb(III) concentration increased again, the biomass of XK8 stabilized at around 0.22 g (Figure 3).

### Biosorption of Cd(II) and Sb(III) Onto *Curvularia coatesiae* XK8

**FIGURE 4:**
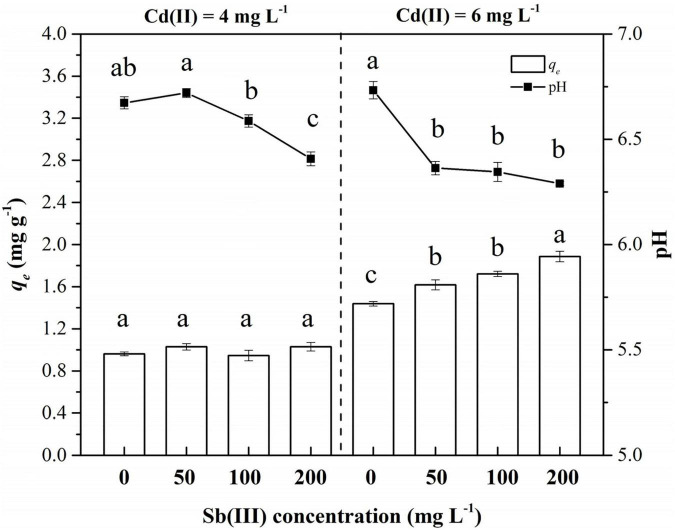
Biosorption of Cd(II) and Sb(III) onto XK8. Letters indicate results of one-way ANOVA. If the letters are completely different, the difference is significant (*p* < 0.05), otherwise the difference is not significant (*p* > 0.05).

The biosorption of *C. coatesiae* XK8 on Cd(II) was affected by Cd(II) concentration, Sb(III) concentration and their interaction (*p* < 0.05) (Table 1). When the initial Cd(II) concentration was 4 mg/L, the removal amount of Cd(II) by XK8 is 0.9627 mg/g. After the addition of Sb(III), the removal amount of Cd(II) by XK8 increased with the increase of Sb(III) concentration (*p* > 0.05). When the Sb(III) concentration reached the maximum, the removal of cadmium by XK8 also reached the maximum, which was 1.0300 mg/g. When the initial cadmium concentration was 6 mg/L, antimony significantly increased the removal of cadmium by XK8 (*p* < 0.05). When the Sb(III) concentration reached the maximum [200 mg/L Sb(III)], the removal amount increased from 1.4383 mg/g to 1.8875 mg/g (Figure 4).

**TABLE 1:** ANOVA results for effects of Cd(II), Sb(III), and their interactions on the biosorption characteristics of XK8.

	Cd(II)	Sb(III)	Cd(II) × Sb(III)
Remove amount (*q*_*e*_)	585.99***	15.24***	10.11***
pH	56.81***	49.42***	17.69***

Values are F and symbols show p, ***p < 0.001.

At the same time, the addition of Sb(III) reduced the pH of the biosorption system (Table 1). When Cd(II) was added alone, the pH of the solution was about 6.70 (Figure 4). The pH value gradually decreased with the increase of Sb(III) concentration. When the initial Cd(II) concentration was 4 mg/L, the pH decreased from 6.67 to 6.41. When the initial Cd(II) concentration was 6 mg/L, the pH decreased from 6.73 to 6.29. Therefore, it was necessary for us to study the effect of pH variation on the biosorption characteristics of XK8.

### The Effect of Environmental Factors on the Biosorption Characteristics

Box-Behnken experimental design (BBD) was used to study the main and interaction effects of different factors. In our study, BBD was applied to establish the combined effect of four selected parameters [A: initial Cd(II) concentration, B: initial Sb(III) concentration, C: biosorption time, D: initial pH of heavy metal solution] on the biosorption process of Cd(II) and Sb(III) on XK8. The experimental results were shown in Supplementary Figure 2 and Table 2. The quadratic polynomial equations were fitted for the biosorption progress.

**TABLE 2:** Experimental design matrix of the Box-Behnken design.

Std	Run	A (mg/L)	B (mg/L)	C (d)	D	Dry weight (g), Y1	pH, Y2	Experimental Cd(II) removal (%), Y3	Experimental Sb(III) removal (%), Y4
28	1	5	60	4	4	0.2203	6.28	73.03	15.94
14	2	5	100	1	4	0.2126	6.13	62.13	27.54
13	3	5	20	1	4	0.1943	6.27	45.01	26.24
22	4	5	100	4	2	0.2482	4	17.61	21.63
7	5	5	60	1	6	0.1849	6.19	47.95	15.94
10	6	6	60	4	2	0.2988	5.02	21.24	27.12
1	7	4	20	4	4	0.3412	6.64	83.08	18.75
16	8	5	100	7	4	0.3651	6.17	81.59	19.67
29	9	5	60	4	4	0.2733	6.44	81.02	15.94
2	10	6	20	4	4	0.3066	6.56	69.41	18.75
27	11	5	60	4	4	0.2825	6.51	84.81	15.94
3	12	4	100	4	4	0.2498	6.26	82.11	20.65
18	13	6	60	1	4	0.1800	6.19	60.84	14.35
8	14	5	60	7	6	0.3405	6.29	74.72	17.54
19	15	4	60	7	4	0.4073	6.54	84.41	17.54
26	16	5	60	4	4	0.2841	6.5	85.17	14.35
23	17	5	20	4	6	0.2624	6.59	79.77	18.75
25	18	5	60	4	4	0.3279	6.5	83.62	15.94
12	19	6	60	4	6	0.2473	6.31	72.48	17.54
15	20	5	20	7	4	0.3262	6.63	83.09	18.75
9	21	4	60	4	2	0.2958	4.51	15.01	28.71
30	22	5	60	4	4	0.2850	6.39	83.15	19.13
20	23	6	60	7	4	0.3817	6.25	77.03	14.35
11	24	4	60	4	6	0.2460	6.38	82.11	12.75
17	25	4	60	1	4	0.1785	6.17	50.00	14.35
24	26	5	100	4	6	0.2616	6.4	82.68	23.60
21	27	5	20	4	2	0.3419	4.75	23.48	26.24
4	28	6	100	4	4	0.2385	6.3	72.87	21.63
5	29	5	60	1	2	0.2080	3.73	13.41	35.09
6	30	5	60	7	2	0.3273	4.6	13.89	31.90


$$Y_{1}=+0.28-5.475E-003A-0.016B+0.082C-0.015D$$

Y2=+6.44+0.011A-0.18B+0.15C+0.96D+0.030AB-0.078AC-0.15AD-0.080BC+0.14BD-0.19CD+0.053A-28.333E-004B-20.19C-20.99D2

Y3=+81.80-1.90A+1.26B+11.28C+27.92D+1.11AB-4.55AC-3.96AD-4.66BC+2.20BD+6.57CD-2.74A-21.21B-212.31C-231.02D2

Y4=+16.21+0.082A+0.60B-1.15C-5.38D+0.25AB-0.80AC+1.59AD-0.097BC+2.36BD+1.20CD-1.02A+23.45B+22.33C+25.27D2

The *p* value of the model was less than 0.0001, and the *p* value of the lack of fit term was 0.2509, indicating that the model lack of fit was not significant, but the regression was significant. The correlation coefficient *R*^2^ is 0.9742, indicating that the equation fitted well. The coefficient of variation (*CV*) was 9.1% (<10%), which means that the Box-Behbken model had good credibility and accuracy. Adeq Precision means the ratio of signal to noise, which should usually be greater than 4.0. The precision of this study was 19.4, indicating that the model can properly reflect the test results (Supplementary Table 2). The analysis of variance showed that there was a high correlation between the predicted value of this study and the measured value, which can be applied to the theoretical prediction of cadmium biosorption by microbial adsorbents.

**FIGURE 5:**
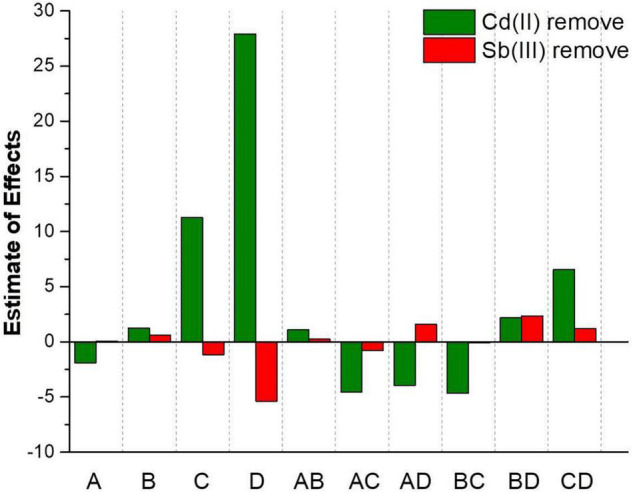
Impact assessment of different factors on Cd(II) and Sb(III) biosorption by XK8.

According to Y_3_, the main effect of Cd(II) initial concentration (−1.90) on the Cd(II) biosorption process was negative, while the main effect of the Sb(III) initial concentration (+1.26), biosorption time (+11.28), and initial pH (+27.92) were positive. Hence, the influence of various factors for the Cd(II) removal rate was initial pH > biosorption time > Cd(III) initial concentration > Sb(III) initial concentration. According to Y_4_, the main effect of the biosorption time (−1.15) and the initial pH (−5.38) on the Sb(III) biosorption process were negative, while the main effect of the Cd(II) initial concentration (+0.082) and the Sb(III) initial concentration (+0.60) were positive. Therefore, the influence of various factors on the Sb(III) removal rate was initial pH > biosorption time > Sb(III) initial concentration > Cd(II) initial concentration. It seemed likely the biosorption of two metal ions may promote each other when Cd(II) and Sb(III) were co-adsorbed. As depicted in Figure 5, it could be clearly observed that the effect of initial pH and biosorption time on the Sb^3+^ biosorption process was opposite the effect on the Cd^2+^ biosorption process. With the conditions of higher pH and longer biosorption time, the Cd^2+^ removal rate increased, but the Sb^3+^ removal rate decreased.

**FIGURE 6:**
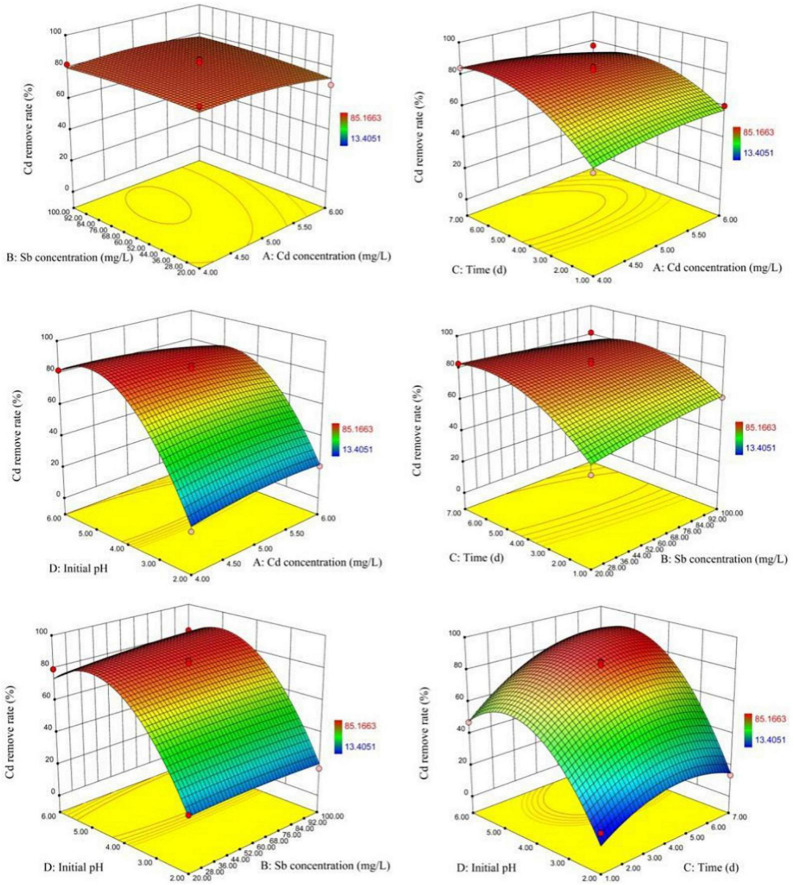
Response surface curves for Cd(II) removal showing the binary interactions.

According to Figure 6, it could be clearly from the 3D graph observed that when the initial pH range was 4–6 and the biosorption time was 4–6 days, the Cd(II) removal rate was significantly improved. It can be seen from the 2D profile curve that in the case of a long biosorption time, a slight increase of the initial pH would lead to a significant increase for the Cd(II) biosorption capacity. This shows that the high initial pH of the heavy metal solution is an important factor for the improvement of biosorption efficiency, and this effect is particularly obvious in the lengthening of biosorption time.

**FIGURE 7:**
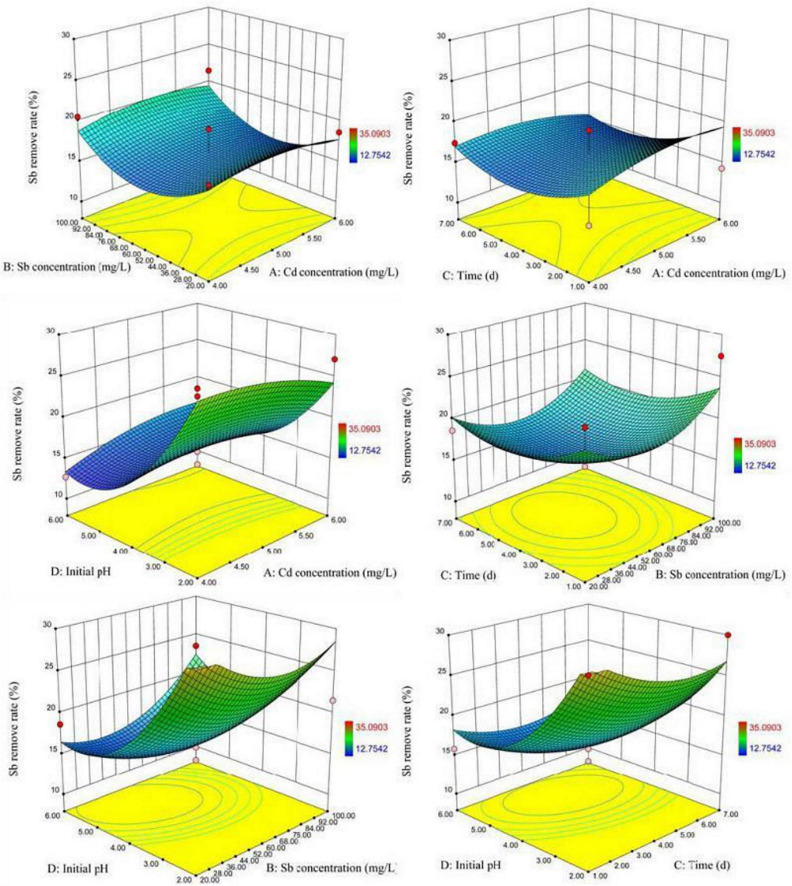
Response surface curves for Sb(III) removal showing the binary interactions.

Figure 7 visually showed the 3D surface response graph and the 2D contour analysis graph of the interaction of the various factors of XK8 adsorbing antimony under the compound condition. The results showed that the interaction of the Cd(II) initial concentration, Sb(III) initial concentration, biosorption time, and initial pH was not significant. When the Sb^3+^ initial concentration was about 4.5–5.5 mg L^–1^, the initial pH was about 2–3, and the biosorption time was about 1–3 days, XK8 has the highest Sb(III) removal rate.

According to SAS analysis, the theoretical maximum removal rates of Cd(II) and Sb(III) were 100 and 24.6%, respectively. The corresponding optimal conditions for removal of Cd(II) and Sb(III) were: the Cd(II) initial concentration was 6 mg L^–1^, the Sb(III) initial concentration was 100 mg L^–1^, biosorption time was 7 days, initial pH was 6 and the Cd(II) initial concentration was 6 mg L^–1^, the Sb(III) initial concentration was 100 mg L^–1^, biosorption time was 7 days, and initial the pH was 5.94. By experimenting with the best removal conditions for Cd(II) and Sb(III), the actual maximum removal rates of Cd(II) and Sb(III) were 67.57 and 16.75% (Table 3).

**TABLE 3:** Theoretical and actual maximum removal rates of Cd(II) and Sb(III) under optimal conditions.

	Optimum process parameters				Predicted (%)	Actual (%)
	A	B	C	D		
Cd(II) remove rate	6	100	7	6	100	67.57
Sb(III) remove rate	6	100	7	5.94	24.5985	16.75

### Functional Groups of *Curvularia coatesiae* XK8 During Cd(II) and Sb(III) Biosorption

**FIGURE 8:**
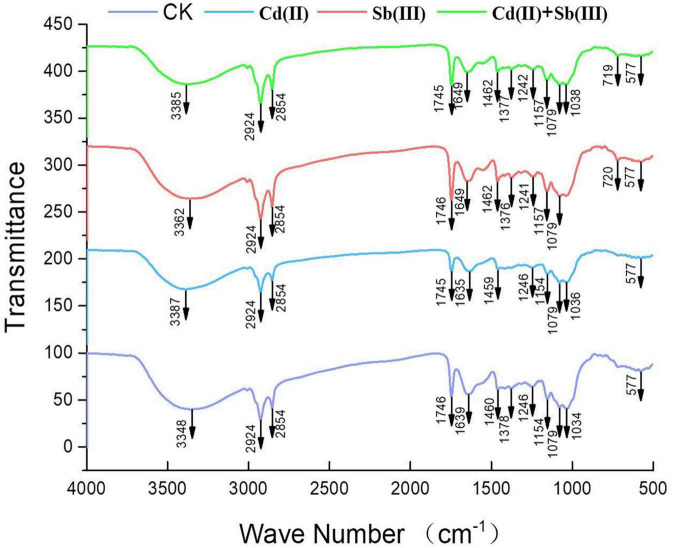
FTIR spectra of XK8 biomass before and after heavy metal loading.

The main functional groups related to the strong peaks observed are identified by previous literature (Xu et al., 2012; Xia et al., 2015; Kumar and Dwivedi, 2019) and listed in Table 4. The peak at 3348 cm^–1^ strongly shifted, indicating –OH stretching vibration and –NH stretching of the protein (Figure 8). The disappeared peak at 1378 cm^–1^ assigned to amide III band represents COO– anions. The disappeared peak at 720 cm^–1^ was attributed to an interaction between Cd(II) and phosphate or sulfur functional groups. Compared CK with the single biosorption and binary biosorption, the shift of Figure 8 suggested that COO– and sulfur groups could be active functional sites for the binding of Sb(III).

**TABLE 4:** Main functional groups of FTIR analysis.

Wavelength (cm^–1^)	Functional group
3300–3400	–OH stretching vibration and –NH stretching of the protein
2924 and 2854	Asymmetric/symmetric stretching vibration of CH2
1746	Stretching of C–O group
1639	C = O stretching vibration and –NH deformation Amide I
1460	C–N stretching in Amide III
1378	Amide III band represents to COO–anions
1246	–SO_3_ groups
1154	Stretching vibration of C–O–C
1079	C–N stretching vibration of amino groups and phosphate groups
1034	C–N stretching vibration of the chitin-chitosan
500–700 (fingerprint zone)	Phosphate or sulfate functional groups

## Discussion

### The Role of Environmental Factors in the Biosorption Process of Fungi

The single biosorption characteristics showed that the initial cadmium concentration in the solution affected the growth and biosorption efficiency of the strain. With the increase of cadmium concentration, the growth and cadmium removal rate of XK8 decreased gradually, indicating that the growth of fungi was inhibited. This may be due to the destruction of antioxidant the enzyme system and genetic material of microorganisms by heavy metal cadmium, and the inhibition of transcription process. When the concentration of cadmium was 1 mM, the removal rate of XK8 was the highest, and decreased with the increase of concentration. This indicated that at low concentration, when the strain provided too many active biosorption sites, Cd in the solution would interact with the binding sites, and the biosorption sites were higher than the ion concentration. However, with the increase of cadmium concentration, the competition of biosorption sites and the decrease of microbial activity might result in the decrease of biosorption rate (Choinska-Pulit et al., 2018).

Biosorption time is one of the important factors in biosorption process (Hassan et al., 2018). For Cd(II), with the increase of biosorption time, the biomass of XK8 increased first and then tended to be flat. When the biosorption time was 1–4 days, the removal rate of cadmium by XK8 increased significantly, which was due to the large number of bioactive biosorption sites on the surface of fungal cells at the initial stage of biosorption, so the biological biosorption rate of metal ions was faster. For Sb(III), the biomass of XK8 first increased and then decreased with the increase of biosorption time. The results showed that antimony had toxic effect on XK8 when the biosorption time was 7 days. Therefore, when the biosorption time was 4–7 days, the removal rate of antimony decreased.

Among the physicochemical factors, pH may be the most important factors. In almost all biosorbent materials (including bacteria, algae, and fungi), the biosorption of heavy metals often shows a strong dependence of pH. The initial pH of solution affects the competition between hydrogen ions and metal ions for biosorption surface active sites, the permeability of cell membrane, the ionization in solution and the activity of functional groups, as well as the functional groups in cell wall and the metabolic activities in cells, which is one of the important factors affecting the biosorption of metal ions (Cai et al., 2018). For Cd(II), the biosorption rate of XK8 for Cd(II) increased significantly when the initial pH was 2–4, and reached the maximum at pH 4. When the initial pH was 4–6, the removal rate of XK8 decreased, but not significantly. For Sb(III), when the initial pH was 2–4, the removal rate of Sb by XK8 decreased significantly. When the initial pH was 4–6, the removal rate of Sb by XK8 had no significant difference. No matter how the pH changed, the removal rate of Sb(III) by XK8 was less than 40%. The low removal rate of antimony by fungal adsorbents was mainly related to the chemical properties of antimony. Antimony was difficult to be adsorbed due to its non-polar characteristics (Li et al., 2016). Sb(III) exists in the form of SbO^+^ [or Sb(OH)^2+^] under very acidic conditions, and in the form of H_2_SbO^3–^ or Sb(OH)^4–^ under weak acidic, neutral, and alkaline conditions (Cai et al., 2018). The functional groups involved in metal absorption and metal chemistry can be used to explain the pH dependence of biosorption efficiency. When the initial pH was low, the pH value of the medium decreased significantly. A large number of H^+^ and H_3_O^+^ ions occupied most of the biosorption sites on the surface of the fungal cell wall. Only a small amount of Cd^2+^ and Sb(OH)^2+^ ions could be adsorbed by fungi. When the initial pH was 4–6, the medium pH of XK8 increased rapidly to about 7.0. At this time, antimony in the solution existed in the form of H_2_SbO^3–^ or Sb(OH)^4–^, the negatively charged antimony and the negative charge on the surface of the biosorbent repulsed each other, and the biosorption rate of antimony decreased significantly.

In the infrared spectrum, the peak at 720 cm^–1^ of the single biosorption system of cadmium disappeared, which may be due to the combination of phosphate or sulfur functional groups and Cd(II). In the infrared spectrum, the peak at 1378 cm^–1^ of the single biosorption system of cadmium disappeared, which may be due to the combination of carboxyl group and Cd(II). In the four groups of biosorption experiments, the peak at 3348 cm^–1^ vibrated, which may be caused by the interaction of –OH stretching vibration and –NH stretching of the protein with Cd(II) and Sb(III). In the four groups of biosorption experiments, the peak at 1241 cm^–1^ vibrated, which may be caused by the interaction of sulfonic acid group with Cd(II) and Sb(III). The peak at 1134 cm^–1^ vibrated, which may be due to the interaction of phosphate group with Cd(II) and Sb(III) (Kumar and Dwivedi, 2019). Studies had shown that microorganisms absorbed heavy metals through cell surface and cell wall. Chitin, glycan, and cellulose in fungal cells all played an important role in metal adhesion. Antimony and cadmium ions were easy to combine with C-O and −NH ligands to absorb heavy metals (Wang and Chen, 2009).

The changes of all environmental factors in this study are discussed in liquid media. Most of the current research on this aspect is mainly studied in liquid. The forms of environmental factors in liquid and soil are generally changed, but the mechanism of changes are still unintelligible (Deng et al., 2012; Wang et al., 2015; Cai et al., 2018; Li et al., 2018).

### The Interaction of Cd(II) and Sb(III) in the Biosorption Process of Fungi

The study on the co-biosorption of Sb and Cd by XK8 showed that the initial pH had the greatest influence on the biosorption of Sb and Cd, followed by the biosorption time, and the influence of both was stronger than that of coexisting ions. The main effect of biosorption time and initial pH on cadmium biosorption was positive, but the main effect on antimony biosorption was negative. When the initial antimony concentration range was 68–76 mg L^–1^, the initial pH range was 4–6, and the inoculation time was 4–6 days, the removal rate of cadmium by XK8 was the highest. When the initial antimony concentration range was 4.5–5.5 mg L^–1^, the initial pH range was 2–3, and the inoculation time was 1–3 days, the removal rate of antimony by XK8 was the highest. pH is an important factor affecting the biosorption of antimony and cadmium. Low pH can promote the biosorption of antimony, while high pH can promote the biosorption of cadmium. Similar results had been found in the studies of biosorption of antimony and cadmium by iron manganese oxides, hematite, kaolinite, and *Bacillus cereus* (Liu et al., 2015; Yang et al., 2018). The study also showed that a shorter biosorption time would promote the biosorption of cadmium, and a longer biosorption time would promote the biosorption of cadmium, which might indicate that XK8 adsorbed antimony before cadmium, but the specific mechanism needs further analysis.

Generally speaking, the presence of other metal ions will affect the biosorption of target ions by microorganisms. For example, the removal rate of Cd(II) by *C. bertholletiae* decreased by 5% after the introduction of competitive ion Pb (II), while the removal rate of Cd(II) by C. *bertholletiae* decreased by 14.3% after the introduction of competitive ion Cu (II). This is because Cd and Cu belong to transition metal ions, while Pb belongs to B-type metal ions. There is no or slight competition between Pb and Cd, which has little effect on each other’s biosorption. However, when Cu and Cd exist at the same time, the removal rate of Cd decreases rapidly (Xu et al., 2017). In the process of simultaneous biosorption of multiple metal ions, the affinity (or removal ability) of different metal ions will also be different. The biosorption capacity of *P. chrysosporium* is stronger than that of Ni when it adsorbs both Cd^2+^ and Ni^2+^ (Noormohamadi et al., 2019). The biosorption capacity of *Bacillus subtilis* for Pb is stronger than that for Sb when both Pb^2+^ and Sb^3+^ are adsorbed at the same time (Cai et al., 2018). When Cu^2+^ and Cr^6+^ are adsorbed at the same time, the biosorption capacity of *P. chrysogenum* for Cr is stronger than that of Cu (Xu et al., 2017). Wang summarized the ability of fungi to adsorb metal ions, the results showed that although the research results of different authors could not be directly compared, some qualitative conclusions could be drawn from these data: the order of fungi biosorption capacity was Cd > Co > Cr > Au≈Cu > Fe > Ni > Th > U > Pb > Hg > Zn. His research also found that *Penicillium* had good biosorption of Cd, Fe, Pb, Th, U, and Zn (Wang and Chen, 2009).

Antimony often coexists with arsenic (As), cadmium (Cd), and other heavy metals, which may further increase the ecological and health risks. Therefore, the study has a good application value for heavy metal removal and pollution control in the coexistence system of a variety of heavy metals. This study showed that Sb(III) could promote the biosorption of Cd(II) on XK8 when Cd(II) and Sb(III) coexist. At present, there are few studies on the removal of heavy metals under the coexistence of antimony and cadmium, and the selected biosorption materials only include Fe–Mn binary oxide (Liu et al., 2015) and ferrihydrite (Yang et al., 2018).

## Conclusion

In this study, a cadmium and antimony tolerant fungus named *C. coatesiae* XK8 was present. This fungus can remove cadmium under a high antimony environment, because it was more capable of removing cadmium than antimony. Its theoretical maximum removal rates of Cd(II) and Sb(III) were 100 and 23.6%, respectively. We conducted experiments on environmental factors that affect the removal efficiency of XK8. The results demonstrated that in the single and binary biosorption systems, the initial heavy metal concentration, the initial pH, and the biosorption time all affected the biosorption of Cd(II) and Sb(III) on XK8. In the environment of strong acid and short biosorption time, XK8 had better biosorption effect on antimony. On the contrary, XK8 had better biosorption effect on cadmium. In the binary biosorption system, Sb(III) can promote the biosorption of Cd(II) on XK8. In the binary biosorption, fungi make the pH of solution slightly acidic, which can promote the biosorption of cadmium. In addition, in the FTIR analysis, it can be inferred that when the biosorption sites of fungi in the process of biosorption of antimony increase, it will also increase the binding of cadmium with functional groups and improve the biosorption rate of cadmium. Considering that the soils of Xikuangshan are in the background of weak acid and high antimony concentration, the environment will promote the biosorption of cadmium on XK8. Therefore, it provides a potential material for the bioremediation of antimony and cadmium pollution in mines.

## Data Availability Statement

The raw data supporting the conclusions of this article will be made available by the authors, without undue reservation.

## Author Contributions

ZD, LC, and XZ designed the sampling and experiments. XZ and YG collected soil samples. ZD and LC analyzed the final data. ZY and XZ acquired funds for this study. ZD wrote the original manuscript. All authors commented on an early draft of the manuscript.

## Conflict of Interest

The authors declare that the research was conducted in the absence of any commercial or financial relationships that could be construed as a potential conflict of interest.

## Publisher’s Note

All claims expressed in this article are solely those of the authors and do not necessarily represent those of their affiliated organizations, or those of the publisher, the editors and the reviewers. Any product that may be evaluated in this article, or claim that may be made by its manufacturer, is not guaranteed or endorsed by the publisher.

## Footnotes

1
http://www.majorbio.com
