# Supplementary material for: Curvularia coatesiae XK8, a Potential Bioadsorbent Material for Adsorbing Cd(II) and Sb(III) Compound Pollution: Characteristics and Effects
Source: Front Microbiol. 2022 Jan 27;12:816312. doi: 10.3389/fmicb.2021.816312 (PMC8828948; doi:10.3389/fmicb.2021.816312)
Supplement: Supplementary file 1 [file Data_Sheet_1.docx]

**Supplementary Information for**

***Curvulariacoatesiae*XK8, a potential** **bioadsorbent material for adsorbing** **Cd(Ⅱ) and Sb(Ⅲ)** **compound pollution: characteristics and effects**

Zhao Di^a,†^, Li Chaoyang^b,†^, Zheng Mengxi^c^, Zhao Yunlin^a,*^, Xu Zhenggang^a,c,*^, Yang Guiyan^c^

*^a^**Hunan Research Center of Engineering Technology for Utilization of Environmental and Resources Plant,* *Central South University of Forestry and Technology,* *Changsha 410004, China*

*^b^ Central South Inventory and Planning Institute of* *National Forestry and Grassland Administration, Changsha 410014, China*

*^c^**Key Laboratory of National Forestry and Grassland Administration on Management of Western Forest Bio-Disaster, College of Forestry, Northwest A & F University, Yangling, 712100, China*

† These authors contributed equally to this work.

* Correspondence author: Zhao Yunlin, zyl8291290@163.com; Xu Zhenggang, [xuzhenggang@nwafu.edu.cn](mailto:xuzhenggang@nwafu.edu.cn)

**Table S1. Soil characteristics of the soil samples .**

| pH | Metal content（mg/kg）  (mean± SD) | | | | | | | | | | |
| --- | --- | --- | --- | --- | --- | --- | --- | --- | --- | --- | --- |
|  | Sb | As | Fe | Zn | Pb | Mn | Cd | Cr | Sn | Cu | Ni |
| 6.24  ±1.32 | 11752.981  ±176.057 | 2538.965  ±63.849 | 35760.939  ±986.685 | 61.749  ±7.097 | 96.312  ±4.081 | 24.170  ±1.1524 | 64.333  ±5.459 | 110.427  ±2.948 | 0.000 | 7.954  ±0.581 | 7.281  ±1.671 |

**Table S2. The initial concentration of heavy metals in the biosorption system.**

| Number | Cd(II) | Sb(Ⅲ) | Number | Cd(II) | Sb(Ⅲ) | Number | Cd(II) | Sb(Ⅲ) |
| --- | --- | --- | --- | --- | --- | --- | --- | --- |
| 1 | 0 | 0 | 8 | 4 | 30 | 14 | 6 | 30 |
| 2 | 2 | 0 | 9 | 4 | 60 | 15 | 6 | 60 |
| 3 | 4 | 0 | 10 | 4 | 100 | 16 | 6 | 100 |
| 4 | 6 | 0 | 11 | 4 | 200 | 17 | 6 | 200 |
| 5 | 8 | 0 | 12 | 4 | 500 | 18 | 6 | 500 |
| 6 | 10 | 0 | 13 | 4 | 1000 | 19 | 6 | 1000 |
| 7 | 16 | 0 |  |  |  |  |  |  |

**Table S3.**Analysis of variance for BBD response surface experiments.

| Source | Sum of squares | *df* | Mean square | *F*-value | *p*-value |
| --- | --- | --- | --- | --- | --- |
| **Dry weight (Y1)** | | | | | |
| Model | 0.088 | 4 | 0.022 | 25.51 | < 0.0001 |
| A | 3.60E-04 | 1 | 3.60E-04 | 4.20E-01 | 0.5239 |
| B | 3.23E-03 | 1.00E+00 | 3.23E-03 | 3.75E+00 | 0.0642 |
| C | 0.082 | 1 | 0.082 | 9.48E+01 | < 0.0001 |
| D | 2.62E-03 | 1 | 2.62E-03 | 3.04E+00 | 0.0934 |
| Residual error | 0.022 | 25 | 8.609E-004 |  |  |
| Lack of fit | 0.016 | 20 | 7.790E-004 | 0.66 | 0.7735 |
| Pure error | 5.944E-003 | 5 | 1.189E-003 |  |  |
| Cor. total | 0.11 | 29 |  |  |  |
| *R2* = 0.8032; *R2*adj = 0.7717 | | | | | |
| **pH (Y2)** | | | | | |
| Model | 19.32 | 14 | 1.38 | 51.73 | < 0.0001 |
| A | 1.41E-03 | 1.00E+00 | 1.41E-03 | 0.053 | 0.8214 |
| B | 4.00E-01 | 1.00E+00 | 4.00E-01 | 14.85 | 0.0016 |
| C | 0.27 | 1 | 2.70E-01 | 10.12 | 6.20E-03 |
| D | 11.12 | 1.00E+00 | 1.11E+01 | 416.79 | < 0.0001 |
| AB | 3.60E-03 | 1 | 3.60E-03 | 0.13 | 0.7185 |
| AC | 0.024 | 1 | 0.024 | 0.9 | 0.3576 |
| AD | 0.084 | 1 | 0.084 | 3.15 | 0.0961 |
| BC | 0.026 | 1 | 0.026 | 0.96 | 0.3428 |
| BD | 0.078 | 1 | 0.078 | 2.94 | 0.107 |
| CD | 0.15 | 1 | 0.15 | 5.56 | 0.0324 |
| A^2 | 0.019 | 1 | 0.019 | 0.72 | 0.4095 |
| B^2 | 4.76E-06 | 1 | 4.76E-06 | 1.79E-04 | 0.9895 |
| C^2 | 0.26 | 1 | 0.26 | 9.61 | 0.0073 |
| D^2 | 6.75 | 1 | 6.75 | 253.03 | < 0.00 |
| Residual error | 0.4 | 15 | 0.027 |  |  |
| Lack of fit | 0.36 | 10 | 0.036 | 4.48 | 0.0557 |
| Pure error | 0.04 | 5 | 8.03E-03 |  |  |
| Cor. total | 19.72 | 29 |  |  |  |
| *R2* = 0.9797; *R2*adj = 0.9608 | | | | | |
| **Cd(Ⅱ) remove rate (Y3)** | | | | | |
| Model | 18567.16 | 14 | 1326.23 | 40.48 | < 0.0001 |
| A | 4.35E+01 | 1.00E+00 | 4.35E+01 | 1.33 | 0.2673 |
| B | 1.91E+01 | 1.00E+00 | 1.91E+01 | 0.58 | 0.4565 |
| C | 1527.54 | 1 | 1.53E+03 | 46.62 | < 0.0001 |
| D | 9354.76 | 1.00E+00 | 9.35E+03 | 285.53 | < 0.0001 |
| AB | 4.91E+00 | 1 | 4.91E+00 | 0.15 | 0.7042 |
| AC | 82.97 | 1 | 82.97 | 2.53 | 0.1324 |
| AD | 62.83 | 1 | 62.83 | 1.92 | 0.1864 |
| BC | 86.77 | 1 | 86.77 | 2.65 | 0.1245 |
| BD | 19.3 | 1 | 19.3 | 0.59 | 0.4547 |
| CD | 172.68 | 1 | 172.68 | 5.27 | 0.0365 |
| A^2 | 51.52 | 1 | 51.52 | 1.57 | 0.229 |
| B^2 | 1.01E+01 | 1 | 1.01E+01 | 3.10E-01 | 0.5879 |
| C^2 | 1038.36 | 1 | 1038.36 | 31.69 | < 0.0001 |
| D^2 | 6597.9 | 1 | 6597.9 | 201.39 | < 0.0001 |
| Residual error | 491.44 | 15 | 32.76 |  |  |
| Lack of fit | 388.41 | 10 | 38.84 | 1.89 | 0.2509 |
| Pure error | 103.02 | 5 | 20.6 |  |  |
| Cor. total | 19058.59 | 29 |  |  |  |
| *R^2^*= 0.9742; *R^2^*_adj_ = 0.9501 | | | | | |
| **Sb(Ⅲ) remove rate (Y4)** | | | | | |
| Model | 697.47 | 14 | 49.82 | 3.14 | 0.0177 |
| A | 8.00E-02 | 1.00E+00 | 8.00E-02 | 5.07E-03 | 0.9442 |
| B | 4.34E+00 | 1.00E+00 | 4.34E+00 | 0.27 | 0.6088 |
| C | 15.79 | 1 | 1.58E+01 | 0.99 | 3.35E-01 |
| D | 347.48 | 1.00E+00 | 3.47E+02 | 21.87 | 0.0003 |
| AB | 2.40E-01 | 1 | 2.40E-01 | 0.015 | 0.9035 |
| AC | 2.54 | 1 | 2.54 | 0.16 | 0.6948 |
| AD | 10.17 | 1 | 10.17 | 0.64 | 0.4362 |
| BC | 0.037 | 1 | 0.037 | 2.35E-03 | 0.962 |
| BD | 22.34 | 1 | 22.34 | 1.41 | 0.2542 |
| CD | 5.72 | 1 | 5.72 | 0.36 | 0.5575 |
| A^2 | 7.09 | 1 | 7.09 | 0.45 | 0.5142 |
| B^2 | 8.16E+01 | 1 | 8.16E+01 | 5.13E+00 | 0.0387 |
| C^2 | 37.16 | 1 | 37.16 | 2.34 | 0.147 |
| D^2 | 190.7 | 1 | 190.7 | 12 | 0.0035 |
| Residual error | 238.32 | 15 | 15.89 |  |  |
| Lack of fit | 226.04 | 10 | 22.6 | 9.2 | 0.0122 |
| Pure error | 12.28 | 5.00E+00 | 2.46 |  |  |
| Cor. total | 935.79 | 29 |  |  |  |
| *R^2^*= 0.7453; *R^2^*_adj_ = 0.5076 | | | | | |


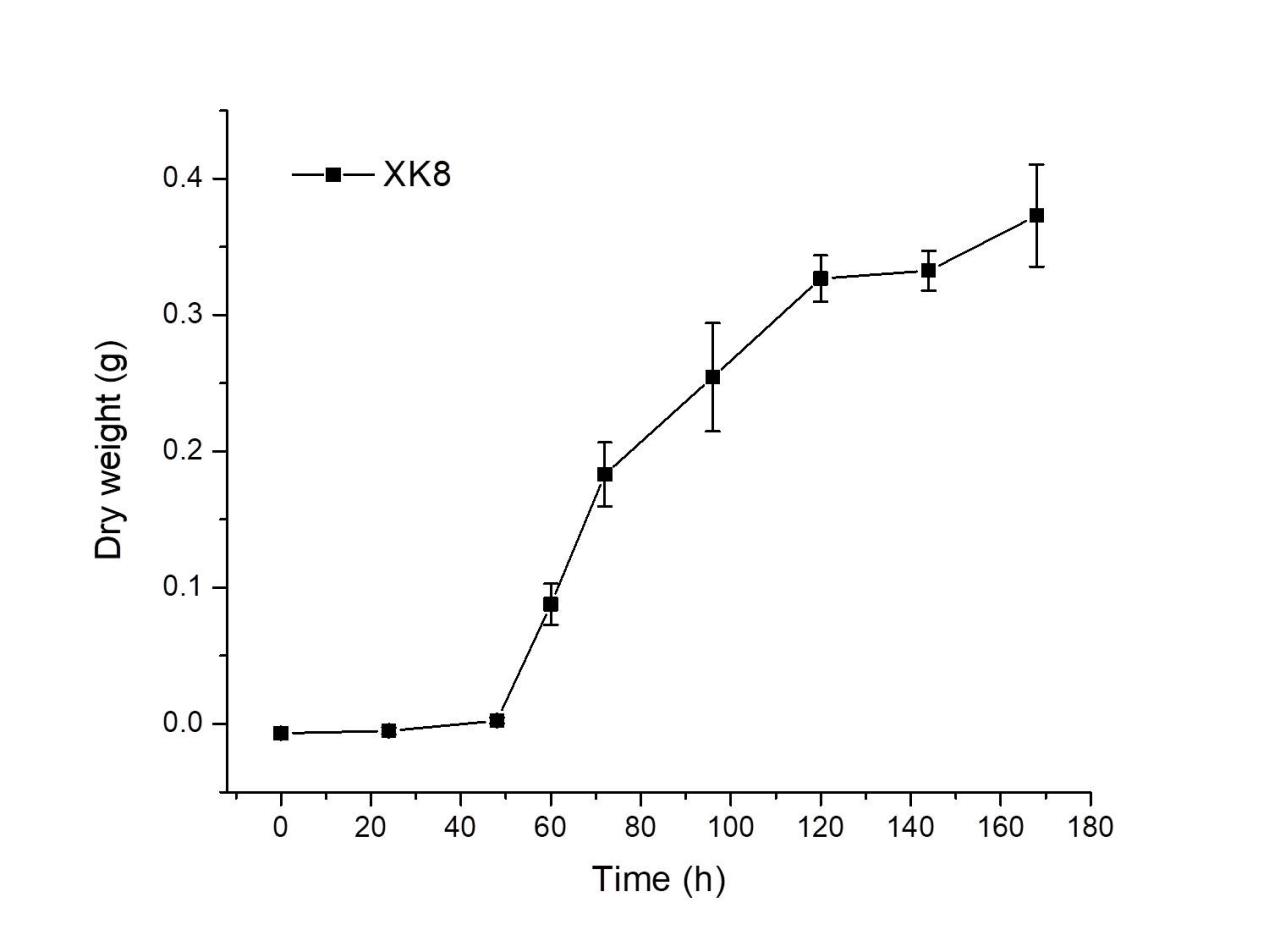


**Figure S1**. Growth curve of XK8.


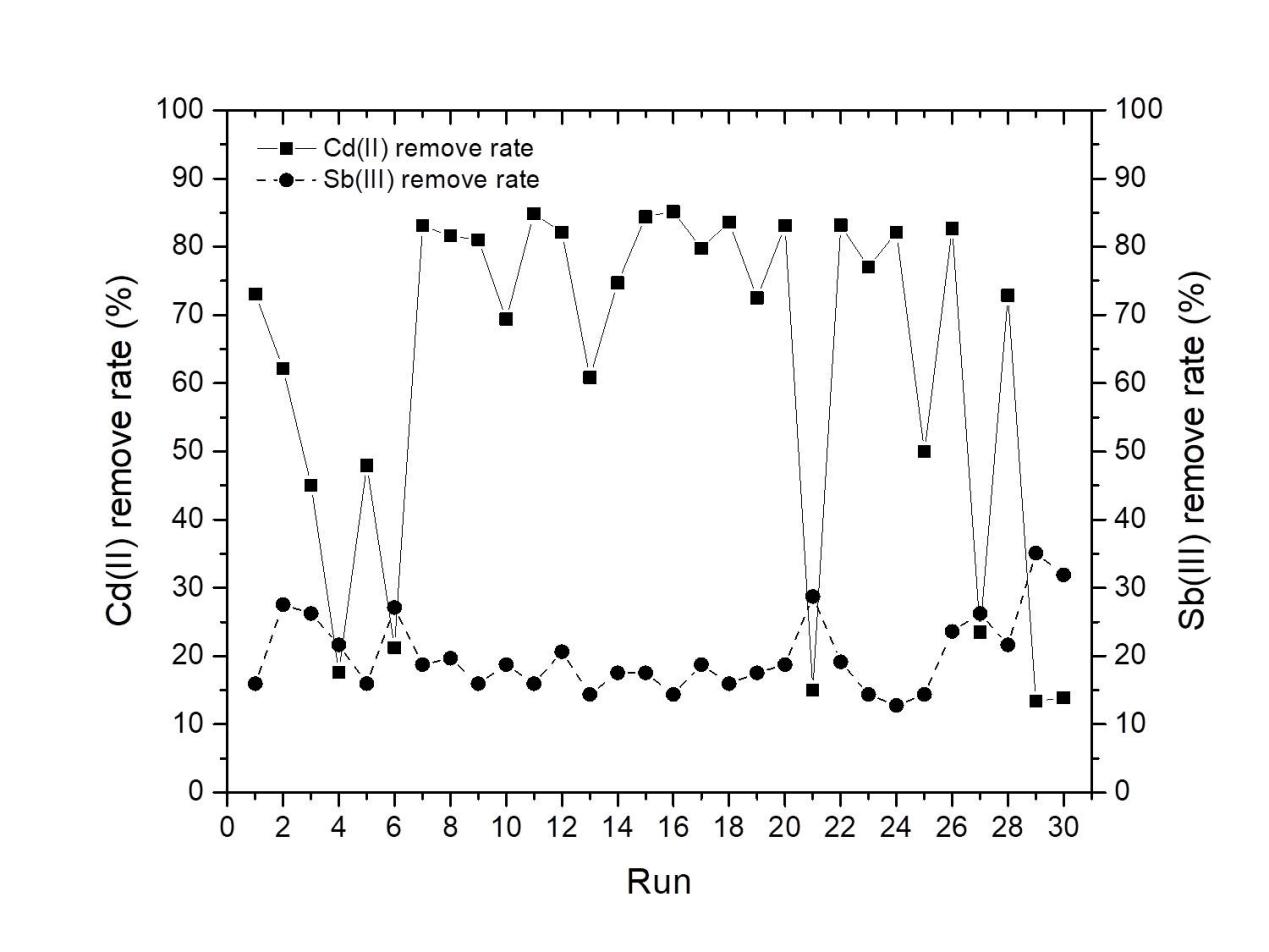


**Figure S2.** Cd(Ⅱ) and Sb(Ⅲ) removal rate by an optimum fungus under various experimental conditions designed by BBD.
